# The Legal Aspects

**Published:** 1920-07-31

**Authors:** 


### THE ADMINISTRATION OF THE LUNACY ACTS.

### The Legal Aspects.

The result of the appeal in the case of *Everett* v. *Ankelsaria and Griffiths*, heard in the Court of Appeal on March 30, is that two of the Lords Justices (Bankes and Scrutton, L.JJ.) decided against the appellant, while the third judge (Atkin, L.J.) was in his favour, and thought a new trial ought to be ordered. The action was for damages for having wrongfully caused the appellant to be confined in a lunatic asylum. The respondent Griffiths was the person who made the reception order, and the respondent Ankelsaria was the doctor who gave the certificate which accompanied the order. Quite exceptional conflicts of view disclosed themselves in the case. When first tried the jury were unable to agree on the question left them by the Lord Chief Justice, as to whether the defendants, who were sued for damages for having unlawfully procured the plaintiff's detention in a lunatic asylum, had exercised reasonable care. Notwithstanding this, the Lord Chief Justice entered judgment for the defendants on the ground that they were acting in a judicial capacity in doing what they did. The majority of the Lords Justices, in dismissing the appeal, thought that Griffiths, who was Chairman of the Board of Guardians, did all that was required of him to fulfil the duty imposed on him by the Lunacy Acts. As to the other defendant, the medical officer who signed the certificate of unsoundness of mind upon which the plaintiff was detained, the Lords Justices thought that it was sufficient if he used ordinary care in what he did, and that the question of whether he also used reasonable skill need not be considered. At the same time, the Judge who gave the leading judgment (Bankes, L.J.) was satisfied on reading the evidence that there was no failure to use reasonable skill on the part of the medical man. The dissentient Judge (Atkin, L.J.) thought that the Chairman of the Board had no judicial privilege in what he did, that there was a duty on the medical man to use a reasonable degree of professional skill as well as care, and that there was evidence of want of care on the part of both defendants.

Although the rights and duties of medical practitioners and others in certifying and taking care of lunatics are now regulated by statute, the opinion expressed by Lord Campbell, C.J., in *Fletcher* v. *Fletcher* (1858, 1 E. & E., 420), is still the governing principle of interpretation, and any violation of the liberty of the subject should be justified on the clearest grounds.

### The Acts of 1890 and 1891.

In framing the procedure to be adopted before a person can be confined as a lunatic the Legislature has undoubtedly been confronted with a difficult and delicate task. The public interest has had to be considered as well as that of the individual alleged to be a lunatic or of unsound mind, and provision has had to be made for dealing with cases of emergency, and for authorising persons, who cannot in many cases have any special knowledge or experience of mental diseases, to take the necessary steps to confine persons or to place them under control as lunatics. The law has been amended from time to time, and is contained in the Lunacy Acts, 1890 and 1891. In speaking of one of the earlier Lunacy Acts, Lord Denman, in Shuttleworth's case (9 Q.B., at p. 658), refers to the protection afforded by that Act as "double—against improper confinement and against danger from a lunatic being unrestrained." Lord Lindley, in *Reg.* v. *Whitfield* (15 Q.B.D., at p. 150), says of the Lunacy Act of 1853: "The statute has given justices of the peace and medical men large powers, but the statute is based upon the theory that they can be trusted. Similar language can be found in other decisions upon the Lunacy Acts, and it is entirely applicable to the Acts of 1890 and 1891, and should be borne in mind in construing those Acts and in endeavouring to ascertain the intention of the Legislature from the language used in them."

It becomes necessary, therefore, to look carefully into the provisions of the Lunacy Acts, to ascertain what duty it is that the Acts cast upon the respondents in reference, in the one case, to the making of the reception order, and, in the other, to the giving of the medical certificate. It is material to notice at this point that the appellant in the court below expressly disclaimed any imputation of want of good faith against either respondent.

### The Procedure Laid Down.

The appellant, throughout the proceedings to which the respondents were parties, was treated as a pauper lunatic, and was dealt with under the sections of the Act of 1890. In the first instance he had been treated as an urgent case under Section 20, and had been removed to the workhouse. It then became the duty of the relieving officer, under Section 14 (2), to give notice that the appellant was deemed to be a lunatic to a justice who had jurisdiction in the place where the appellant resided. When this was done the section directs the justice to require the relieving officer to bring the alleged lunatic before him, and when this is done Section 16 provides for the subsequent procedure. The section requires the justice before whom a pauper who is alleged to be a lunatic is brought (*a*) to call in a medical parctitioner, (*b*) to examine the alleged lunatic, and (*c*) to make such inquiries as he thinks advisable; and it provides, that if upon such examination or other proof the justice is satisfied that the alleged lunatic is a lunatic and a proper person to be detained, he may, provided that the medical practitioner who has been called in signs a medical certificate with regard to the lunatic, by order direct the lunatic to be received and detained in an institution for lunatics named in the order. Each of the respondents acted under this section, and each was called to account by the appellant for what he did.

### The Case of Griffiths.

Let us apply these principles, then, to the case of Griffiths. He was not a Justice of the Peace. In making the detention order he was acting under the special authority conferred upon him as chairman of the Islington Guardians by the Lord Chancellor, under section 25 of the Lunacy Act, 1891. This section is curiously worded. It provides that every order signed under the authority so given "shall have effect as if made by a justice of the peace under the principal Act." The section is silent on the point whether a chairman of guardians, acting under an authority so given, is himself to be considered as if he were a justice of the peace. The appellant urged that the effect of the section is to give the reception order the same force and effect as if made by a justice of the peace, but that it does not clothe a chairman of guardians with the same immunity as a justice of the peace acting under the principal Act from any consequences of making the order. This argument was rejected as being not well founded. The section must be read as meaning that the

**The Administration of the Lunacy Acts**—(*continued*).

same consequences are to follow the making of the order as if it had been made by a justice of the peace under the principal Act. If, therefore, the respondent Griffiths was honestly satisfied (as it was admitted that he was) that the plaintiff was at the time when he made the reception order a lunatic and a proper person to be detained, he was justified under the statute in making the detention order, and the Court thought it immaterial whether or not he used reasonable care in arriving at his decision.

The Doctor's Case.

As regards the case of the doctor, the Lord Chief Justice decided this branch of the case against the appellant, presumably on the authority of the decision of the Court of Appeal in *Thompson* v. *Schmidt* (56 J.P., 212). Having regard to the view of the facts which was taken by the Court in that case, it was distinguishable from the present on the ground that the defendant there was a mere volunteer, and his so-called certificate was a mere statement of opinion in writing, whereas in the present case the respondent Anklesaria was called in to make the examination of the appellant which is prescribed by the statute, and to give the certificate without which the reception order could not have been made. The point for consideration in the doctor's case, therefore, was whether a medical man who is called in to make an examination of an alleged lunatic under Section 16 of the Lunacy Act, 1890, and who, after such examination gives a certificate under the statute, is under any and what duty to the person whom he examines and certifies.

Dr. Anklesaria was called in because he was the doctor of the workhouse. The Court could not accept the view that in those circumstances he escaped all responsibility to the appellant, merely because he could in no sense be said to have been retained or employed by or on behalf of the appellant. Dr. Anklesaria, by undertaking the examination of the appellant, did come under a duty to him, though the extent and limits of that duty must be ascertained by reference to the terms of the statute by which the examination is prescribed. Section 16 says nothing as to what the medical practitioner shall do when called in by the justice, except that he may sign a certificate. If the person who makes the examination is under any duty at all to the person examined by him, that duty must include a duty to act in good faith, and any wilful misstatement is, by the terms of the statute, in itself a misdemeanour. It must include, also, a duty to act with reasonable care in making the examination, because in a matter of such importance the Legislature could not have contemplated any other examination than one conducted with such care as in the circumstances of each case could come up to the standard of what was reasonable.

The Meaning of the Statute.

Whether any duty of acting with reasonable skill as well as with reasonable care is imposed by the statute is a matter on which there is considerable doubt. To adopt the view that a medical man who is called in by a magistrate to express an opinion on a person who is alleged to be a lunatic may be liable in damages for making an incorrect diagnosis, though he may have acted in perfect good faith and used all the care at his command, is to adopt a view which may render the working of the Act in many cases extremely difficult, if not impossible—a result which the Legislature may well have intended to avoid. The appellant has not made out any case of want of skill against Dr. Anklesaria. There was no evidence that Dr. Anklesaria failed to use reasonable care and such skill as he possessed in his examination of the appellant, and in arriving at the conclusion expressed in the certificate. In these circumstances, the appeal in his case also failed, and was dismissed, with costs.

